# European Bat Lyssavirus Type 2 RNA in *Myotis daubentonii*

**DOI:** 10.3201/eid1207.060287

**Published:** 2006-07

**Authors:** Nicholas Johnson, Philip R. Wakeley, Sharon M. Brookes, Anthony R. Fooks

**Affiliations:** * Veterinary Laboratories Agency, Addlestone, Surrey, United Kingdom

**Keywords:** European bat lyssavirus, rabies, quantitative PCR, neurotropism, dispatch

## Abstract

Organ distribution of European bat lyssavirus type 2 viral RNA in its reservoir host, *Myotis daubentonii* (Daubenton's bat), was measured with a novel quantitative reverse transcription–polymerase chain reaction assay. High levels of genomic RNA were found in the brain and were also detectable in the tongue, bladder, and stomach.

Bat-mediated rabies has been reported in Europe for more than 50 years. Two variants or genotypes are now recognized that are distinct from rabies viruses of terrestrial mammals and new world bats (*1*). These are known as European bat lyssaviruses (EBLVs). A third lyssavirus, West Caucasian bat lyssavirus, has been isolated in eastern Europe (*2*). EBLV type 1 (EBLV-1) is found throughout mainland Europe and principally associated with the Serotine bat (*Eptesicus serotinus*) (*3*). EBLV-2 is found in *Myotis* bats (*Myotis daubentonii* [Daubenton's bat] and *Myotis dasycneme*) and has been identified in 3 locations in Europe: the Netherlands, the United Kingdom, and Switzerland (Table 1).

**Table 1:** Reports of European bat lyssavirus type 2 (EBLV-2) in Europe, 1985–2004*

Year	County	Description
1985†	Finland	30-year-old man admitted to department of neurology, Helsinki University Central Hospital, with ascending paralysis and radiating pain in right arm and neck; became agitated with hyperexcitability, hyperventilation, and spasms the following day; died 20 days after admission. Rabies diagnosis confirmed by FAT and MIT.
1986	Denmark	Rabies in pond bat (*Myotis dasycneme*).
1986	Denmark	Rabies in Daubenton's bat (*M. daubentonii*).
1986	Germany	Rabies in Daubenton's bat.
1987	Denmark	Rabies in Daubenton's bat.
1987†	The Netherlands	Virus isolated from pond bat in Wommels.
1987†	The Netherlands	Virus isolated from pond bat in Tjerkwerd.
1987	The Netherlands	Virus isolated from pond bat.
1989†	The Netherlands	Virus isolated from pond bat in Andijk.
1992†	Switzerland	Daubenton's bat found hanging on grill of ventilation shaft during daylight hours in Fribourg. Bat was weak, unable to fly, and died shortly afterwards. Rabies diagnosis confirmed by FAT and MIT.
1993†	The Netherlands	Virus isolated from pond bat in Roden.
1993†	Switzerland	Virus isolated from Daubenton's bat in Versoix.
1996†	United Kingdom	Sick Daubenton's bat found in cellar of public house in Newhaven bit a pregnant woman while it was being cared for in bat hospital. Bat deteriorated rapidly. Diagnosis confirmed by FAT, RTCIT, MIT, and RT-PCR.
2002†	United Kingdom	Juvenile female Daubenton's bat brought onto property adjoining Lancashire canal. Bat was in distress with wing damage; was treated for >7 weeks before signs of agitation and vocalization developed; became aggressive and tried to bite handler. Bat died 6 days after symptoms developed. Diagnosis confirmed by FAT, RTCIT, MIT, and RT-PCR.
2002†	Switzerland	Rabies in Daubenton's bat in Geneva.
2002†	United Kingdom	55-year-old man admitted to Dundee hospital with acute hematemesis and upper limb parasthesia; became aggressive and required sedation on day 5; died on day 14. Man had history of exposure to bats in United Kingdom; postmortem PCR on saliva detected EBLV-2. Virus recovered from brain tissue after autopsy.
2003† (bat 696/04)	United Kingdom	Adult male Daubenton's bat found Sep 2003 after flying into tree in daylight near Bury in Lancashire. Bat was cared for by volunteers and took water but attempted to bite carpet when placed there to feed. Bat died and was frozen until diagnosis was made Oct 2004.
2004† (bat 603/04)	United Kingdom	Grounded juvenile female Daubenton's bat was found in Staines and cared for by volunteers. Its condition was poor and it displayed signs of aggression and lethargy. Diagnosis confirmed by FAT, RTCIT, MIT, and RT-PCR.

*FAT, fluorescent-antibody test; MIT, mouse inoculation test; RTCIT; rabies tissue culture infection test; RT-PCR, reverse transcription–polymerase chain reaction. †Virus identity confirmed by genomic sequence analysis.

Two reports detail isolation of EBLV-2 from humans who died of rabies encephalitis in Finland and the United Kingdom (*4**,**5*). In addition, 4 isolations from Daubenton's bat have been reported in the United Kingdom since 1996. Seroprevalence studies suggest that EBLV-2 is maintained at certain sites in the United Kingdom at low levels (*6*). However, the small number of bats infected with EBLV-2 and the nocturnal habits of insectivorous bats have hampered attempts to understand the distribution, prevalence, and transmission of the virus. Biting by Daubenton's bats was suspected in the 2 human cases from Finland and the United Kingdom because both persons had handled this species before symptoms developed. However, like rabies virus, EBLV-2 does not persist in the environment outside of an infected host, and alternative routes of infection should be considered. Investigation of the second bat detected viable EBLV-2 in the brain, and genomic RNA in the heart, stomach, tongue, intestine, liver, and kidney by using a sensitive nested reverse transcription–polymerase chain reaction (RT-PCR) (*7*). This approach was unable to quantify viral RNA within particular tissues. Since this study, 2 additional cases have occurred in the United Kingdom (Table 1). The investigation and quantification of viral load within the infected host could provide evidence for release of virus and methods of transmission.

## The Study

In 2004, two EBLV-2 cases were identified in Daubenton's bats (Table 1). A diagnosis of EBLV-2 infection was confirmed on brain samples with a fluorescent-antibody test, the mouse inoculation test, and a rapid TaqMan assay (*8*). Attempts to culture EBLV-2 from organs in both cases failed because of cytotoxicity of the samples, which destroyed the cell monolayer. Sample dilution reduced the cytotoxic effects of the sample on the cell monolayer (used for virus isolation) and enabled the development of small foci of infection (bat 603/04). Heminested RT-PCR detected virus RNA in brain, tongue, thyroid gland, and bladder after the first round of amplification, and in salivary gland, heart, lung, intestine, and stomach after the second round of amplification. We suspect that inappropriate storage of bat 696/04 in a freezer with repeated freezing and thawing before submission resulted in inactivation of virus in this sample. Heminested RT-PCR detected virus RNA in samples of brain and stomach after the first round of PCR, and in samples of tongue, intestine, liver, and kidney after the second round of amplification.

An EBLV-2-specific real-time PCR was developed to measure virus genome to quantify the potential viral RNA load within organs. Analysis was only attempted on those organs with sufficient RNA within the sample (Figure). Primers EBLVNa (5´-CCTGGCAGATGATGGGAC-3´) and EBLVNb (5´-GCCTTTTATCTTGGATCACT-3´) are located within the nucleoprotein gene and amplify a 221-bp target. An amplified product from a previous case (*5*) was purified by using the RNeasy kit (Qiagen, Valencia, CA, USA) and quantified with a NanoDrop WD-1000 spectrophotometer (NanoDrop Technologies, Inc., Wilmington, DE, USA). This procedure enabled the absolute number of copies of the amplicon to be calculated by its approximate molecular weight and Avogadro's number, as previously described (*9*).

**Figure:**
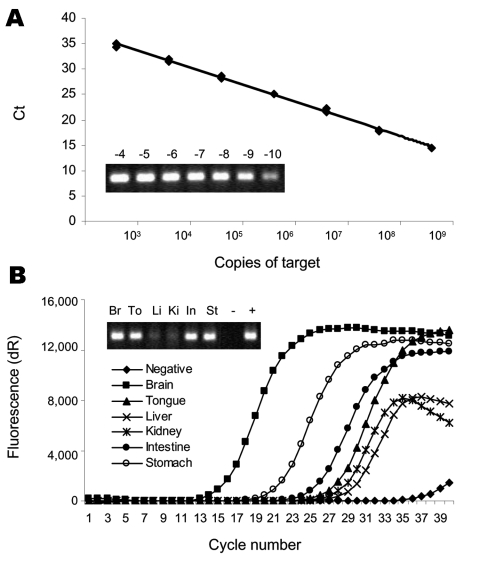
Results of quantitative polymerase chain reaction (PCR) of viral genome copies within organ samples taken from 2 Daubenton's bats infected with European bat lyssavirus type 2 (EBLV-2). A) Standard curve of duplicate dilutions of known quantities of EBLV-2 amplicon. A 20% (vol/vol) sample (10-fold dilution series) separated by electrophoresis on a 1% agarose gel is shown in the inset. Ct, threshold value. B) Real-time PCR amplification of EBLV-2 genomic RNA from organ samples from a bat (696/04) in Lancashire, United Kingdom infected with EBLV-2. dR, fluorescence change generated by a baseline-corrected algorithm calculated from every amplification cycle. A 20% (vol/vol) sample of each amplification separated by electrophoresis on a 1% agarose gel is shown in the inset. Br, brain; To, tongue; Li, liver; Ki, kidney; In, intestine; St, stomach; -, negative sample (no template); +, positive sample from a previous human case (*5*).

RNA was isolated from each organ with Trizol (Invitrogen, Carlsbad, CA, USA) and quantified. Dilutions were made to either 0.25 μg/μL (bat 603/04) or 1 μg/μL (bat 696/04) to standardize the quantity of RNA used for reverse transcription. Primer EBLVNa was used for cDNA synthesis from the genomic (negative) sense strand as previously described (*10*). All PCRs were performed by using SYBR Green JumpStart Taq ReadyMix (Sigma, Saint Louis, MO, USA) and an MX3000P real-time thermal cycler (Stratagene, La Jolla, CA, USA). A dilution series of the control amplicon was amplified simultaneously with the organ samples to create a standard curve for comparison of the threshold value (Ct) with target copy number (Figure, panel A).

A representative plot of amplification curves from organ samples taken from bat 696/04 is shown in the Figure, panel B, with 10 μL of product separated by electrophoresis on a 1% agarose gel included for comparison. The quantitative results for viral RNA load for both bats are shown in Table 2. In both cases, the brain had the highest viral genome load. Virus RNA was consistently detected in the tongue, intestine, and stomach. EBLV-2 was also found in the bladder of bat 603/04 but not in the kidney of bat 696/04 from which the bladder was not recovered because of carcass decomposition. Virus was not detected in the liver of either bat.

**Table 2:** Quantification of European bat lyssavirus type 2 genome copies in organs of 2 naturally infected Daubenton's bats*

	Genome copies/μg total RNA	
Organ	Bat 603/04	Bat 696/04
Brain	204,000,000	640,000,000
Tongue	292,800	136,533
Liver	61,760	37,800
Bladder	839,680	ND
Kidney	ND	87,933
Intestine	277,067	680,667
Stomach	380,133	10,586,667

*ND, not done.

## Conclusions

The detection and quantification of EBLV-2 RNA in bat organs by real-time PCR show the potential distribution of this virus. The choice of organ tested in both cases was severely limited by degradation of the carcass before investigation. Furthermore, live virus could not be recovered from many organs because of cytotoxicity of the samples and virus degradation caused by repeated freezing and thawing.

Viable virus was recovered from the brain of bat 603/04. Since the brain is the main site of EBLV-2 replication, this finding suggests that the virus displays a similar neurotropism to classical rabies virus. Rabies virus, especially in the late stages of disease, disseminates from the brain to other innervated sites within the host (*11*). For EBLV-2, the tongue was consistently found to contain detectable levels of viral RNA in this study and a previous study (7). Genomic RNA was also found in the stomach and intestines of 3 bats investigated (this study and [7]). All of these organs are highly innervated tissues, although virus RNA in the stomach could result from swallowing virus.

Dissemination of rabies virus to the salivary glands and subsequent virus shedding enables transmission through biting. Detection of EBLV-2 RNA in the tongue of infected bats leads us to conclude that transmission of EBLV-2 may occur through biting. However, since EBLV-2 genome was detected in a bladder sample, we cannot exclude the possibility of virus release from urine. In future cases, where possible, organs such as the salivary glands and lungs should be examined to provide further evidence for the route of virus transmission between bats.
